# Supplementary material for: RSK1 vs. RSK2 Inhibitory Activity of the Marine β-Carboline Alkaloid Manzamine A: A Biochemical, Cervical Cancer Protein Expression, and Computational Study
Source: Mar Drugs. 2021 Sep 7;19(9):506. doi: 10.3390/md19090506 (PMC8467814; doi:10.3390/md19090506)
Supplement: Supplementary file 1 [file marinedrugs-19-00506-s001.zip › marinedrugs-1305520-supplementary materials.pdf]

Supporting information

RSK1 vs RSK2 inhibitory activity of the marine β-carboline alkaloid Manzamine A: a biochemical, cervical cancer protein expression and computational docking study

Alejandro M.S. Mayer[1*], Mary L. Hall[1], Joseph Lach[1], Jonathan Clifford[1], Kevin Chandrasena[1], Caitlin Canton[1], Maria Kontoyianni[2], Yeun-Mun Choo[3], Dev Karan[4], and Mark T. Hamann[5]

[1] Department of Pharmacology, College of Graduate Studies, Midwestern University, 555 31st Street, Downers Grove, Illinois 60515, USA; E-Mails: mhall1@midwestern.edu (M.L.H.); joseph.m.lach@gmail.com (J.L.); clifford07@gmail.com (J.C.); kschandras@gmail.com (K.C.); canto030@umn.edu (C.C.)
[2] Department of Pharmaceutical Sciences, Southern Illinois University Edwardsville, Edwardsville, Illinois 62026-2000, USA; E-Mail: mkontoy@siue.edu
[3] Department of Chemistry, University of Malaya, Kuala Lumpur 50603, Malaysia; E-Mail: ymchoo@um.edu.my
[4] Laboratory Medicine, Department of Pathology, Medical College of Wisconsin, Milwaukee, WI; E-Mail: dkaran@mcw.edu
[5] Department of Drug Discovery and Biomedical Sciences and Public Health, Colleges of Pharmacy and Medicine, Medical University of South Carolina, Charleston, South Carolina. 29425, USA; E-Mail: hamannm@musc.edu

* Author to whom correspondence should be addressed; E-Mail: amayer@midwestern.edu
Tel.: (630) 515-6951; Fax: (630) 515-6295.

List of figures

1) **Figure S1:** MZA (blue) and ATP (orange) is shown docked to MAPK-activated protein kinase-1α (binding pocket is shown as gray mesh) (MAPKAP-K1α/RSK1) (PDB ID: 2Z7Q)
2) **Figure S2:** MZA (blue) and ATP (orange) is shown docked to serum and glucocorticoid-induced kinase (binding pocket is shown as gray mesh) (SGK) (PDB ID: 2R5T)
3) **Figure S3:** MZA (blue) and ATP (orange) is shown docked to lymphocyte kinase (binding pocket is shown as gray mesh) (LCK) (PDB ID: 3MPM)
4) **Figure S4:** MZA (blue) and ATP (orange) is shown docked to mitogen and stress-activated protein kinase-1 (binding pocket is shown as gray mesh) (MSK1) (PDB ID: 3KN5)
5) **Figure S5:** MZA (blue) and ATP (orange) is shown docked to glycogen synthase kinase-3β (binding pocket is shown as gray mesh) (GSK3β) (PDB ID: 1Q5K)
6) **Figure S6:** MZA (blue) and ATP (orange) is shown docked to stress-activated protein kinase-2α (binding pocket is shown as gray mesh) (SAPK2α/P38) (PDB ID: 5OMH)
7) **Figure S7:** MZA (blue) and ATP (orange) is shown docked to protein kinase B (binding pocket is shown as gray mesh) (PKBα) (PDB ID: 3CQW)
8) **Figure S8:** MZA (blue) and ATP (orange) is shown docked to c-Jun N-terminal kinase (a binding pocket is shown as gray mesh) (JNK1α1) (PDB ID: 1UKI)
9) **Figure S9:** MZA (blue) and ATP (orange) is shown docked to p70 ribosomal protein S6 kinase (binding pocket is shown as gray mesh) (S6K1) (PDB ID: 3A60)
10) **Figure S10:** MZA (blue) and ATP (orange) is shown docked to dual tyrosine phosphorylated and regulated kinase 1A (binding pocket is shown as gray mesh) (DYRK1A) (PDB ID: 2VX3)
11) **Figure S11:** MZA (blue) and ATP (orange) is shown docked to casein kinase-2 (binding pocket is shown as gray mesh) (CK2) (PDB ID: 1J91)
12) **Figure S12:** MZA (blue) and ATP (orange) is shown docked to checkpoint kinase-1 (binding pocket is shown as gray mesh) (CHK1) (PDB ID: 3U9N)
13) **Figure S13:** MZA (blue) and ATP (orange) is shown docked to C-terminal Src kinase (binding pocket is shown as gray mesh) (CSK) (PDB ID: 1BYG)
14) **Figure S14:** MZA (blue) and ATP (orange) is shown docked to MAPK kinase (mitogen-activated protein kinase) (binding pocket is shown as gray mesh) (MKK1) (PDB ID: 1S9J)
15) **Figure S15:** MZA (blue) and ATP (orange) is shown docked to cyclic AMP-dependent protein kinase (binding pocket is shown as gray mesh) (PKA) (PDB ID: 2JDS)
16) **Figure S16:** MZA (blue) and ATP (orange) is shown docked to protein kinase C (binding pocket is shown as gray mesh) (PKCα) (PDB ID: 3IW4)
17) **Figure S17:** MZA (blue) and ATP (orange) is shown docked to casein kinase-1 (binding pocket is shown as gray mesh) (CK1) (PDB ID: 1CSN)
18) **Figure S18:** MZA (blue) and ATP (orange) is shown docked to MAPK-activated protein kinase-2 (binding pocket is shown as gray mesh) (MAPKAP-K2 (RSK-2)) (PDB ID: 3G51)

19) **Figure S19:** MZA (blue) and ATP (orange) is shown docked to mitogen-activated protein kinase (binding pocket is shown as gray mesh) (MAPK2/ERK2) (PDB ID: 2OJJ)
20) **Figure S20:** MZA (blue) and ATP (orange) is shown docked to NIMA-related protein kinase 2α (binding pocket is shown as gray mesh) (NEK2α) (PDB ID: 5M53)
21) **Figure S21:** MZA (blue) and ATP (orange) is shown docked to AMP-activated protein kinase (binding pocket is shown as gray mesh) (AMPK) (PDB ID: 2YA3)
22) **Figure S22:** MZA (blue) and ATP (orange) is shown docked to stress-activated protein kinase-4 (binding pocket is shown as gray mesh) (SAPK4/P38δ) (PDB ID: 5EKN)
23) **Figure S23:** MZA (blue) and ATP (orange) is shown docked to phosphorylase kinase (binding pocket is shown as gray mesh) (PHK) (PDB ID: 2Y7J)
24) **Figure S24:** MZA (blue) and ATP (orange) is shown docked to stress-activated protein kinase-3 (binding pocket is shown as gray mesh) (SAPK3/P38γ) (PDB ID: 1CM8)
25) **Figure S25:** MZA (blue) and ATP (orange) is shown docked to 3-phosphoinositide-dependent protein kinase-1 (binding pocket is shown as gray mesh) (PDK1) (PDB ID: 5LVL)
26) **Figure S26:** MZA (blue) and ATP (orange) is shown docked to Rho-dependent protein kinase (binding pocket is shown as gray mesh) (ROCK-II) (PDB ID: 6ED6)
27) **Figure S27:** MZA (blue) and ATP (orange) is shown docked to cyclin-dependent kinase 2-cyclin A complex (binding pocket is shown as gray mesh) (CDK2/cyclin A) (PDB ID: 4ez7)
28) **Figure S28.** ^{1}H NMR of Manzamine A (CDCL3, 600 MHz)
29) **Figure S29.** ^{13}C NMR of Manzamine A (CDCL3, 150 MHz)
30) **Figure S30.** 2D-COSY Spectrum Manzamine A (CDCL3, 600 MHz)
31) **Figure S31.** 2D edited g-HSQC Spectrum of Manzamine A (CDCL3, 600 MHz**)**

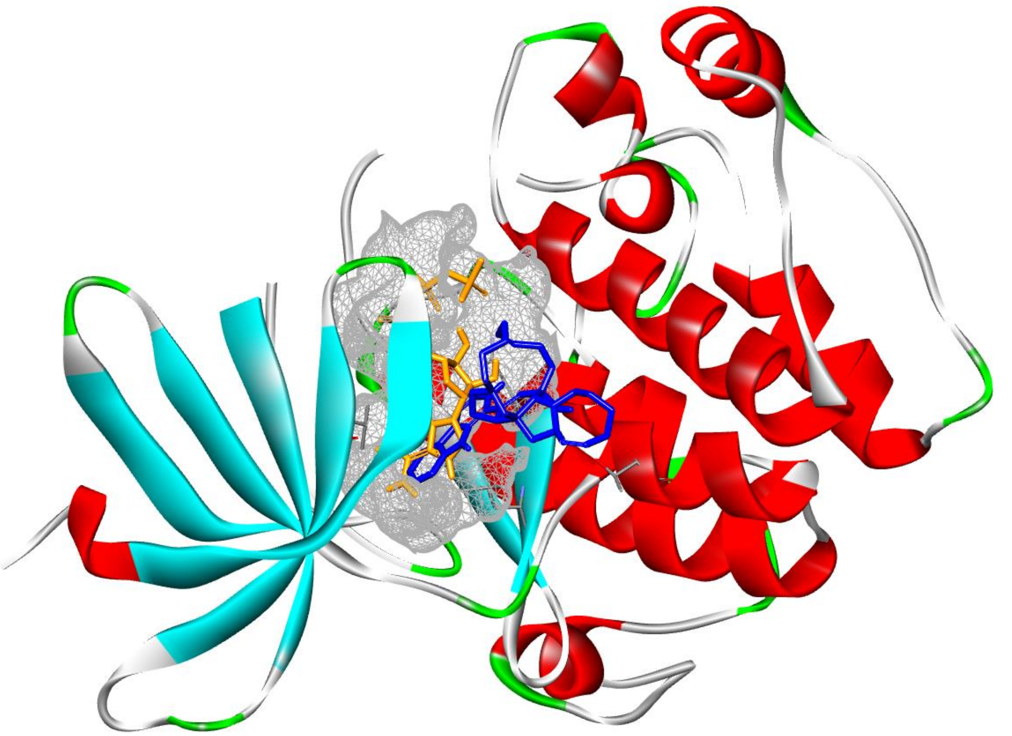

Figure S1: MZA (blue) and ATP (orange) is shown docked to MAPK-activated protein kinase-1α (binding pocket is shown as gray mesh) (MAPKAP-K1α) (PDB ID: 2Z7Q)

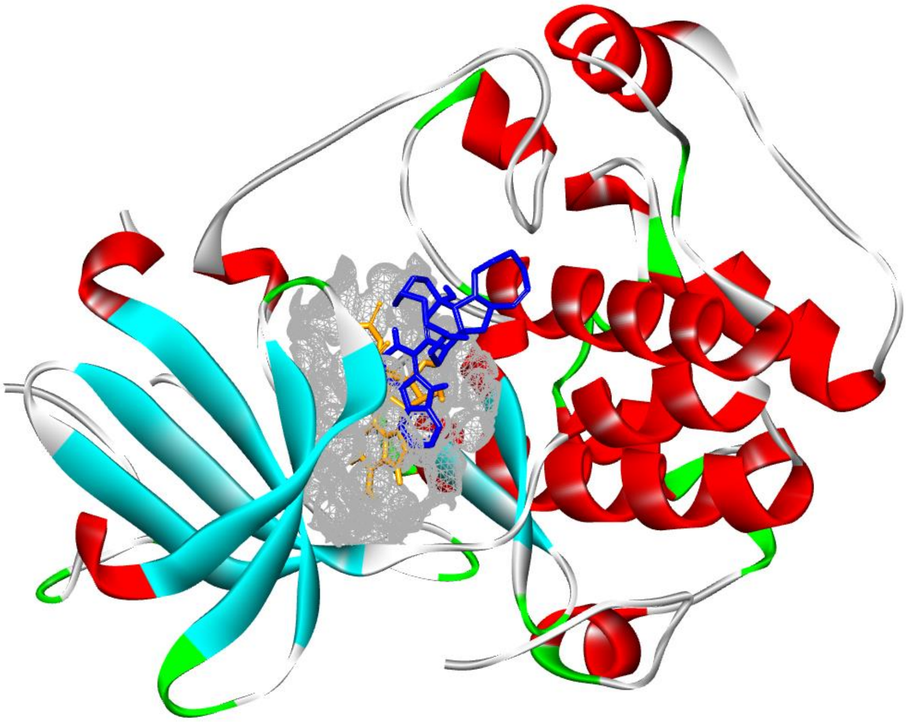

Figure S2: MZA (blue) and ATP (orange) is shown docked to serum and glucocorticoid-induced kinase (binding pocket is shown as gray mesh) (SGK) (PDB ID: 2R5T)

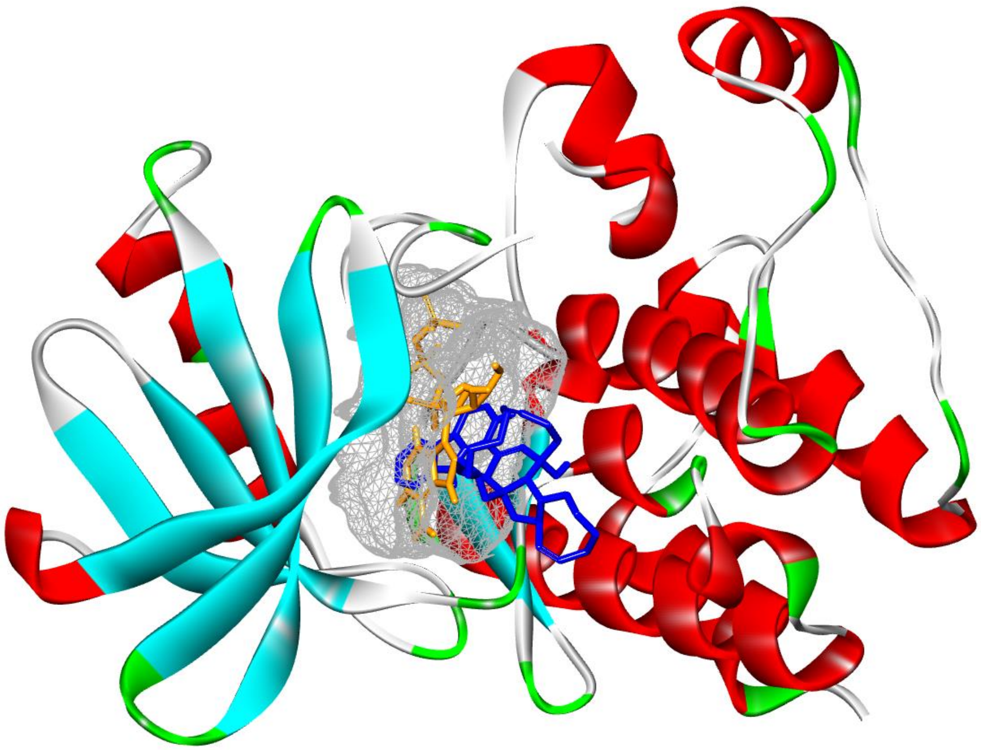

Figure S3: MZA (blue) and ATP (orange) is shown docked to lymphocyte kinase (binding pocket is shown as gray mesh) (LCK) (PDB ID: 3MPM)

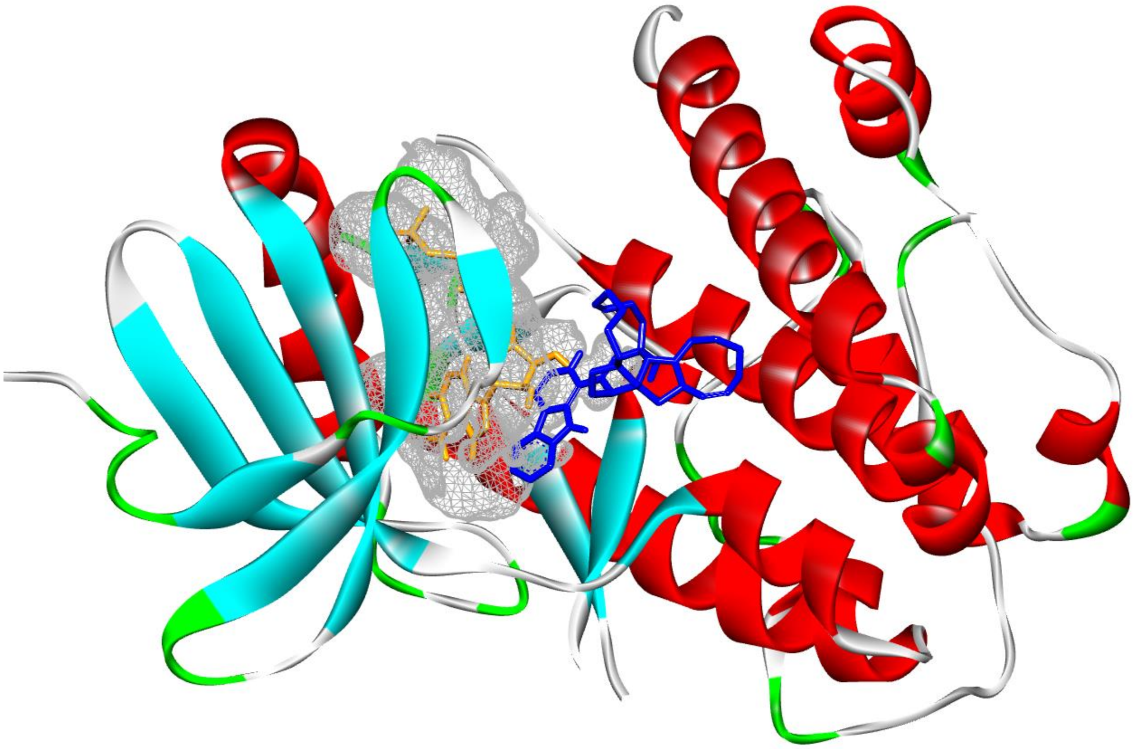

Figure S4: MZA (blue) and ATP (orange) is shown docked to mitogen and stress-activated protein kinase-1 (binding pocket is shown as gray mesh) (MSK1) (PDB ID: 3KN5)

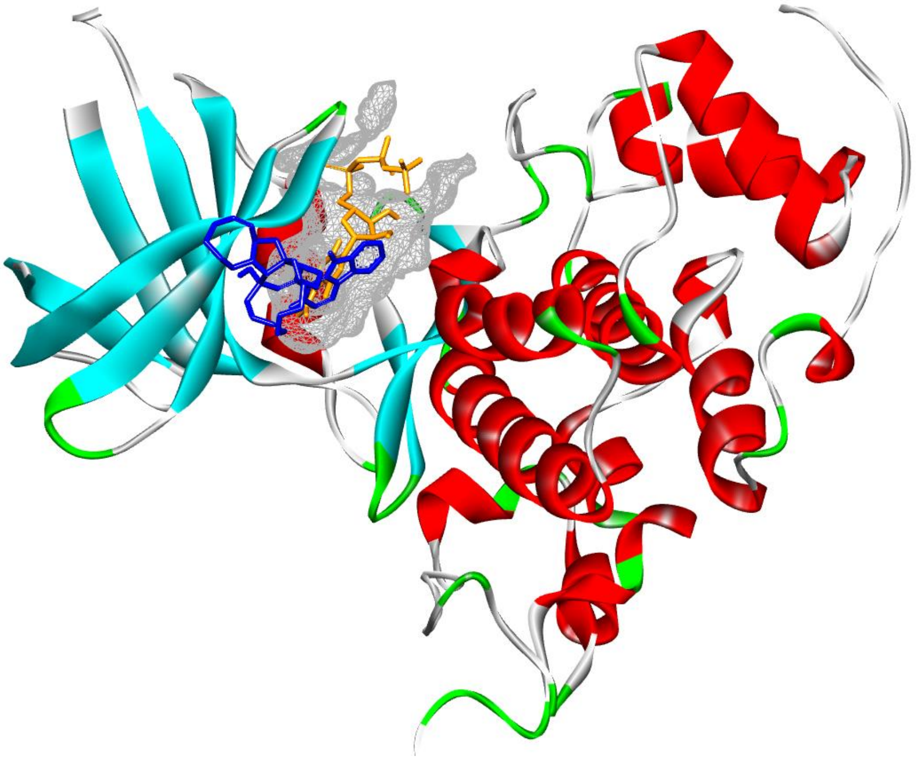

Figure S5: MZA (blue) and ATP (orange) is shown docked to glycogen synthase kinase-3β (binding pocket is shown as gray mesh) (GSK3β) (PDB ID: 1Q5K)

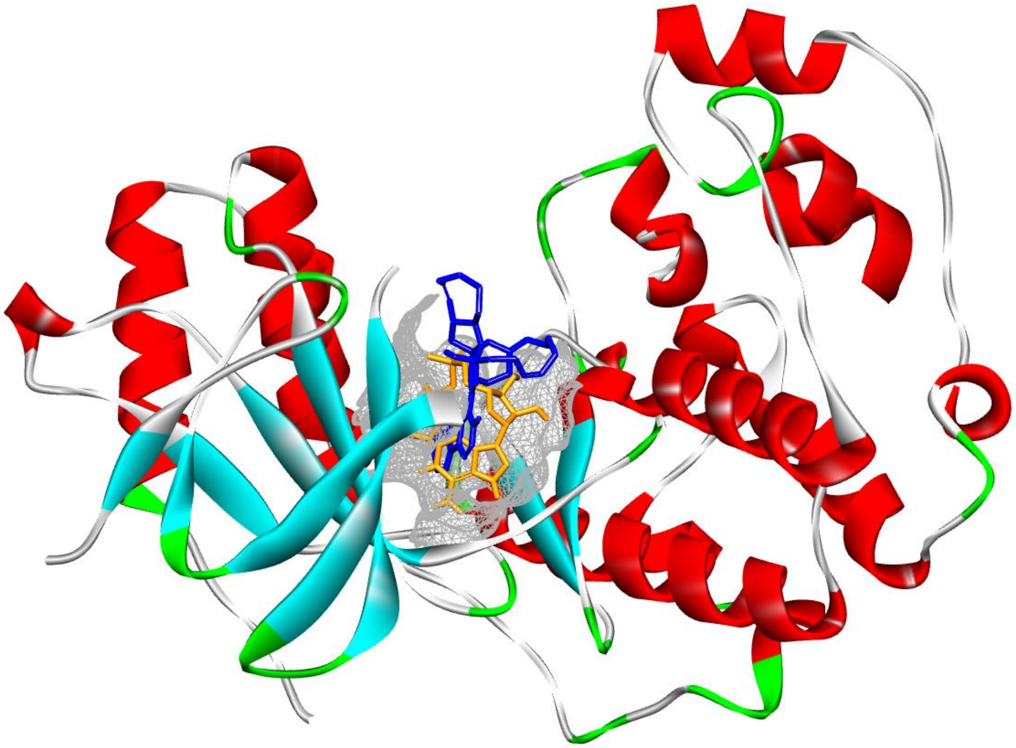

Figure S6: MZA (blue) and ATP (orange) is shown docked to stress-activated protein kinase-2α (binding pocket is shown as gray mesh) (SAPK2α/P38) (PDB ID: 5OMH)

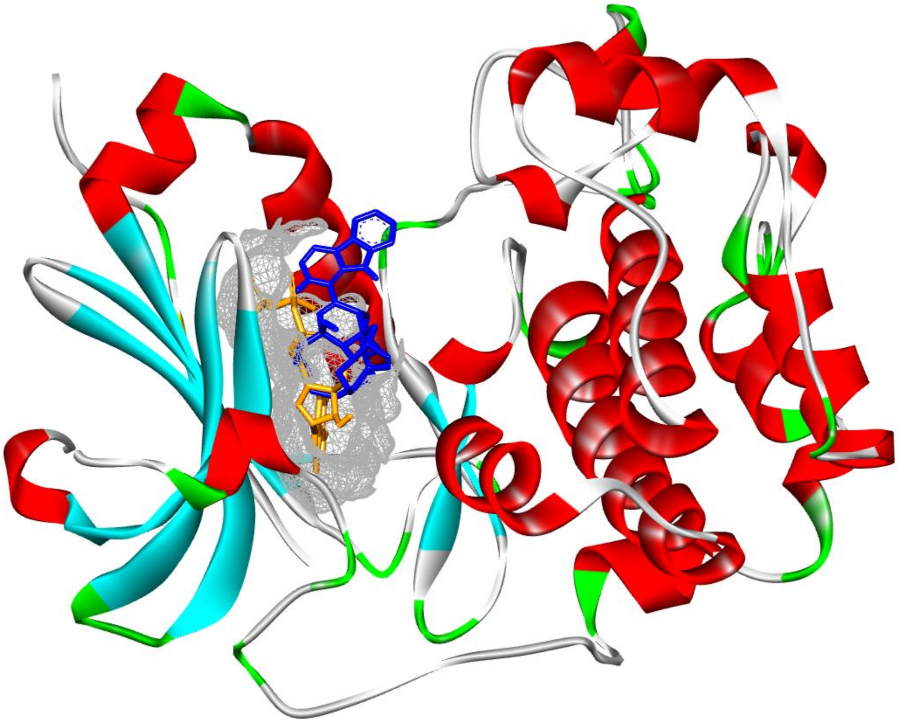

Figure S7: MZA (blue) and ATP (orange) is shown docked to protein kinase B (binding pocket is shown as gray mesh) (PKBα) (PDB ID: 3CQW)

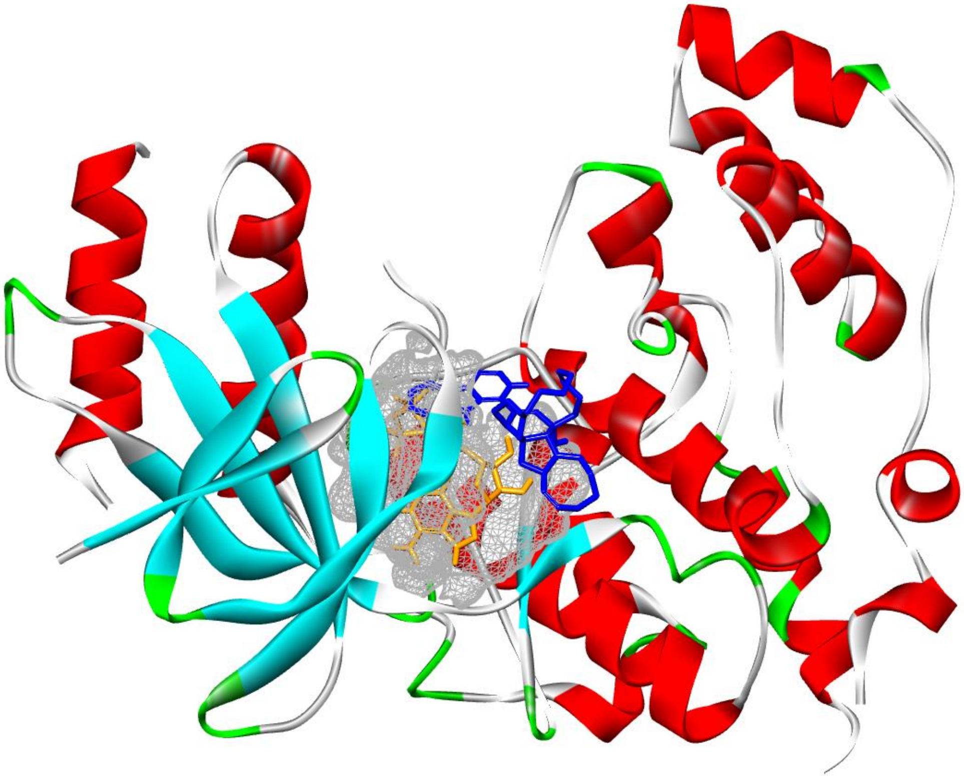

Figure S8: MZA (blue) and ATP (orange) is shown docked to c-Jun N-terminal kinase (binding pocket is shown as gray mesh) (JNK1α1) (PDB ID: 1UKI)

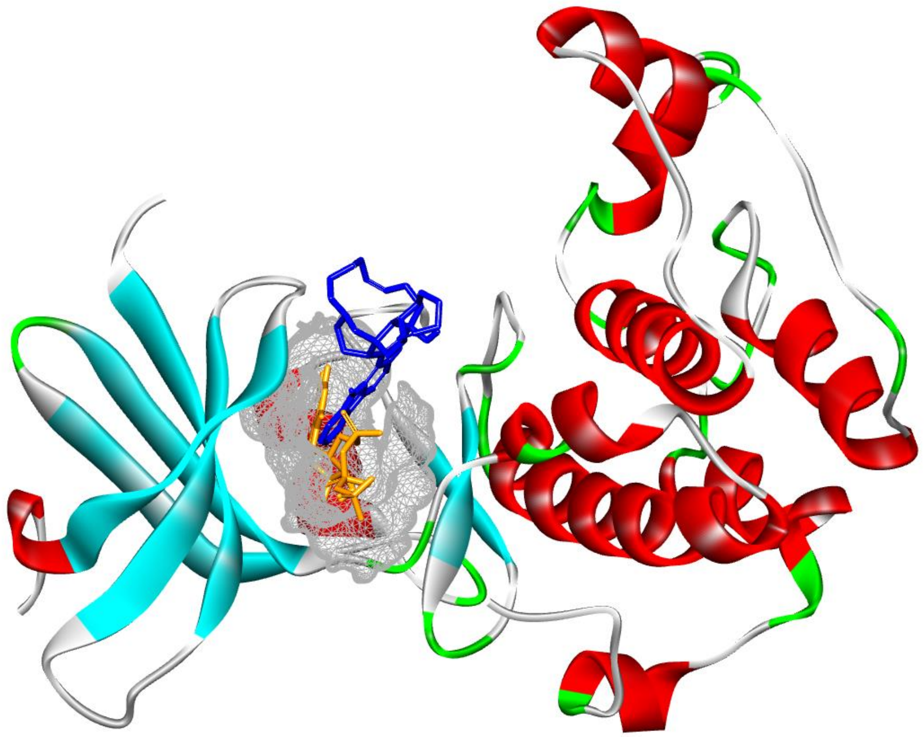

Figure S9: MZA (blue) and ATP (orange) is shown docked to p70 ribosomal protein S6 kinase (binding pocket is shown as gray mesh) (S6K1) (PDB ID: 3A60)

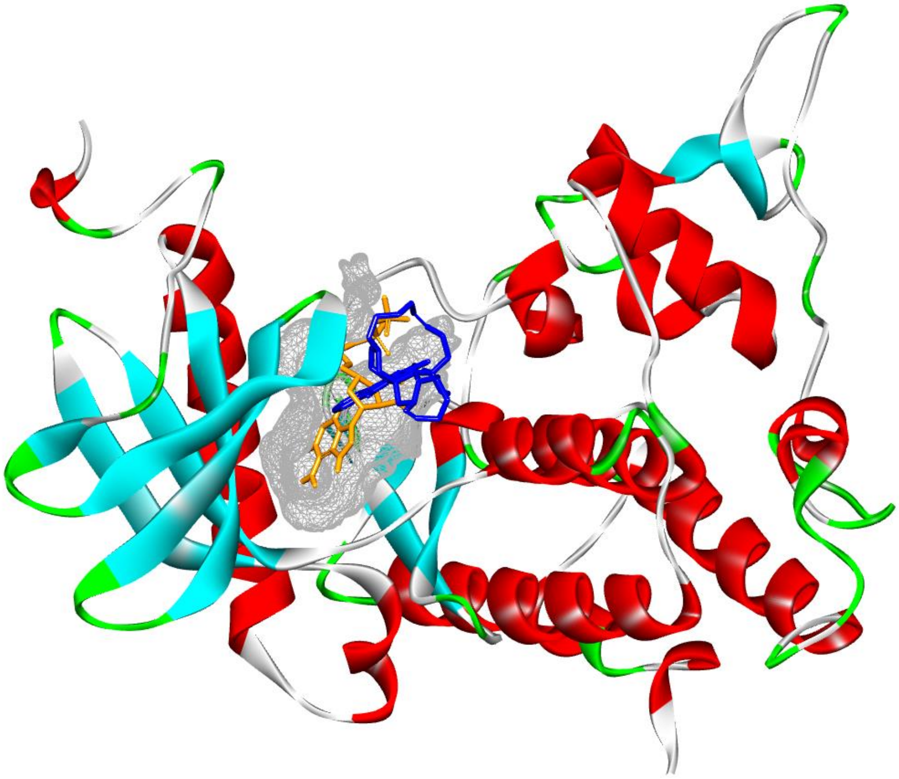

Figure S10: MZA (blue) and ATP (orange) is shown docked to dual tyrosine phosphorylated and regulated kinase 1A (binding pocket is shown as gray mesh) (DYRK1A) (PDB ID: 2VX3)

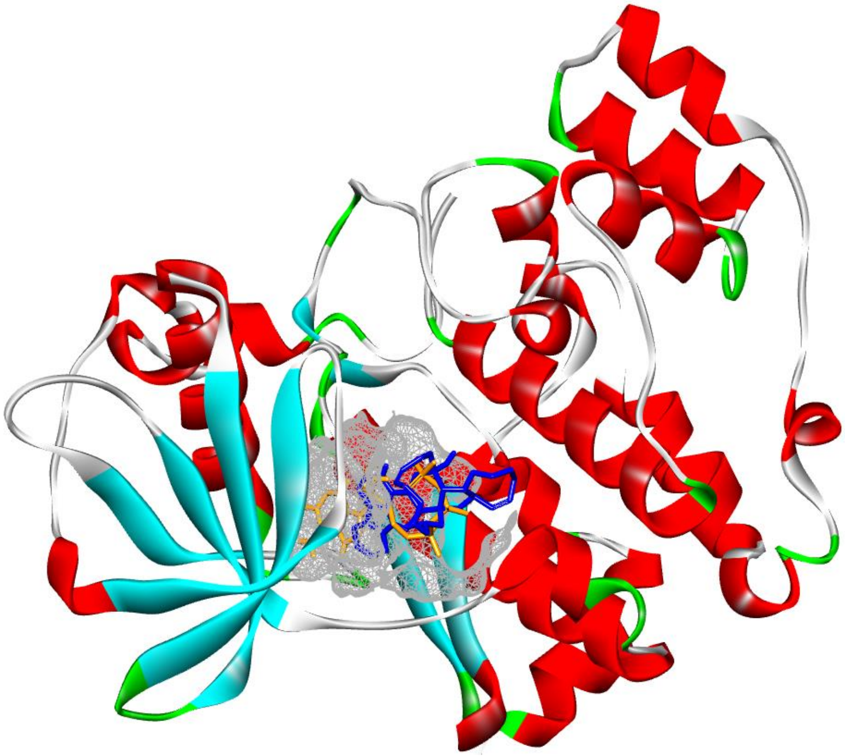

Figure S11: MZA (blue) and ATP (orange) is shown docked to casein kinase-2 (binding pocket is shown as gray mesh) (CK2) (PDB ID: 1J91)

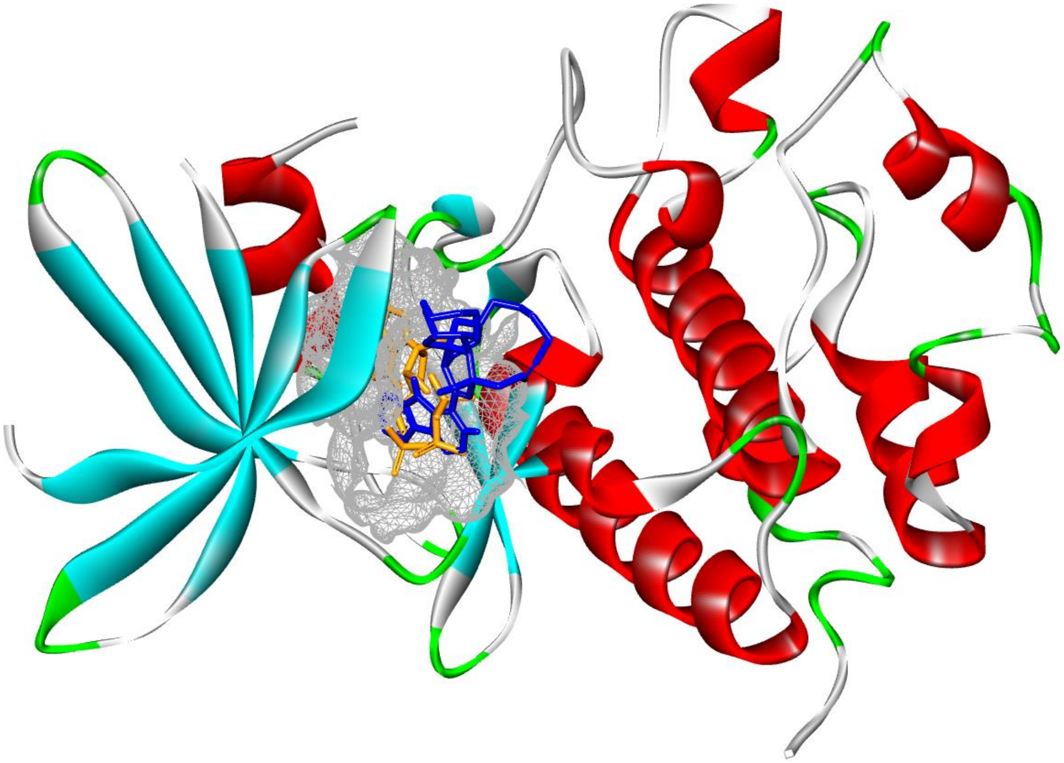

Figure S12: MZA (blue) and ATP (orange) is shown docked to checkpoint kinase-1 (binding pocket is shown as gray mesh) (CHK1) (PDB ID: 3U9N)

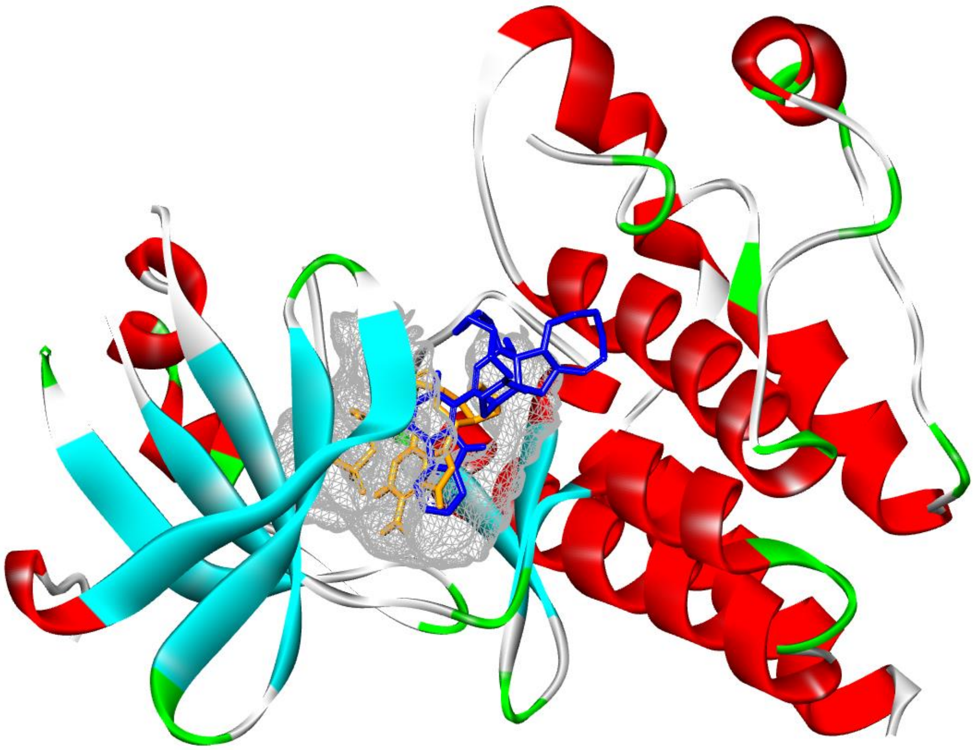

Figure S13: MZA (blue) and ATP (orange) is shown docked to C-terminal Src kinase (binding pocket is shown as gray mesh) (CSK) (PDB ID: 1BYG)

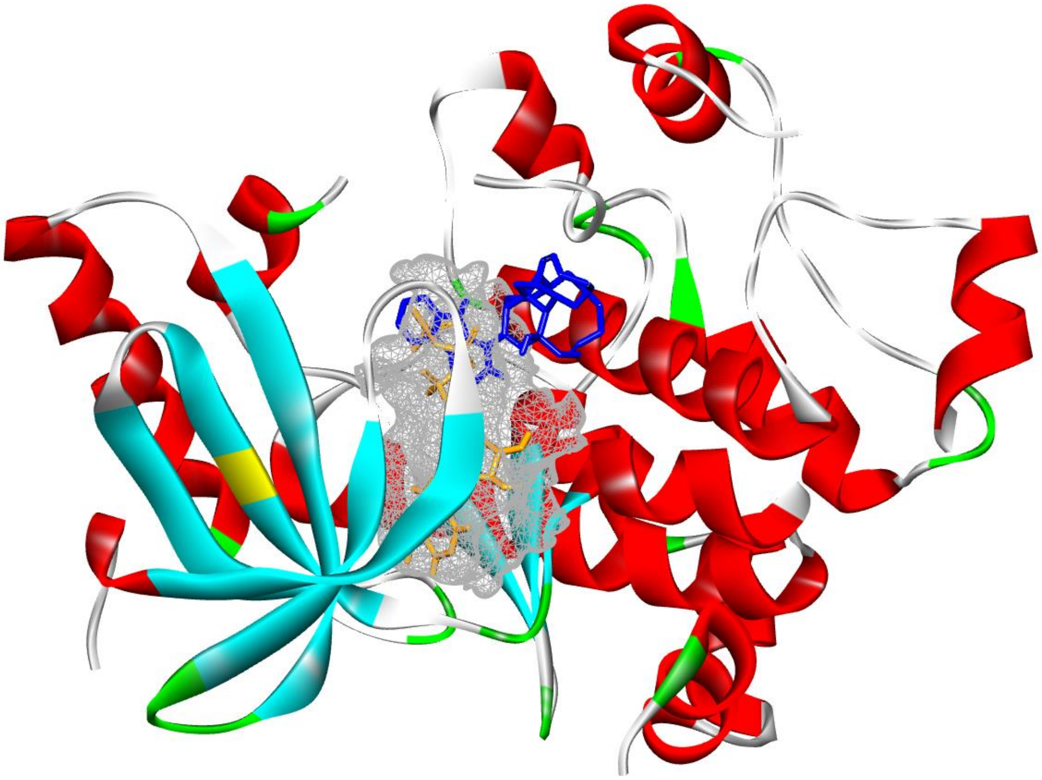

Figure S14: MZA (blue) and ATP (orange) is shown docked to MAPK kinase (mitogen-activated protein kinase) (binding pocket is shown as gray mesh) (MKK1) (PDB ID: 1S9J)

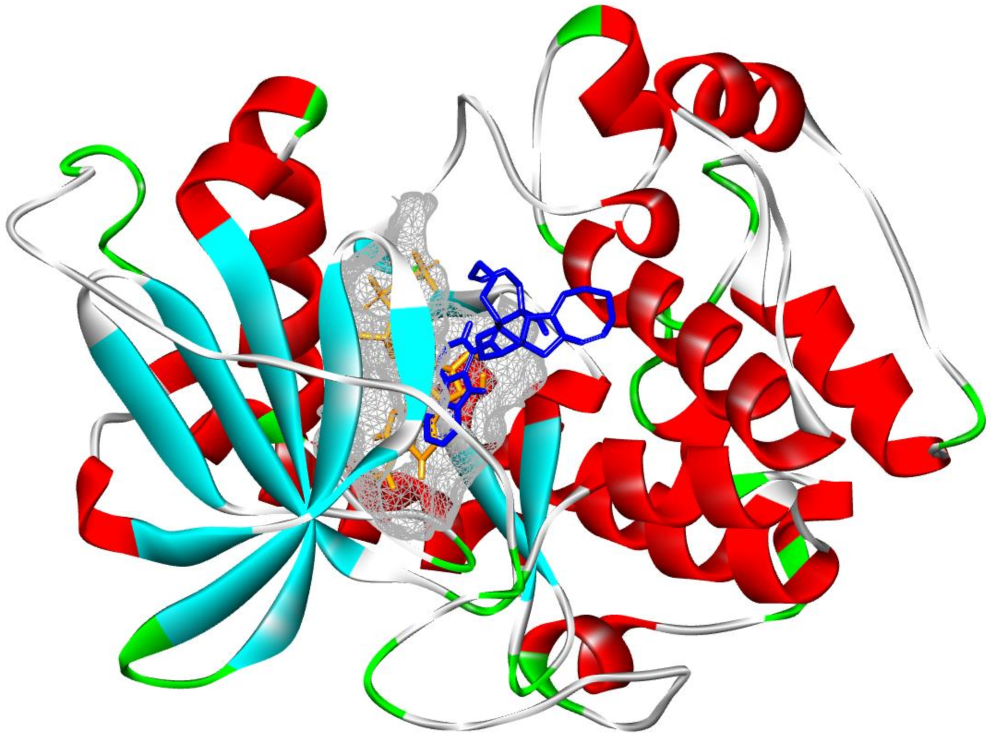

Figure S15: MZA (blue) and ATP (orange) is shown docked to cyclic AMP-dependent protein kinase (binding pocket is shown as gray mesh) (PKA) (PDB ID: 2JDS)

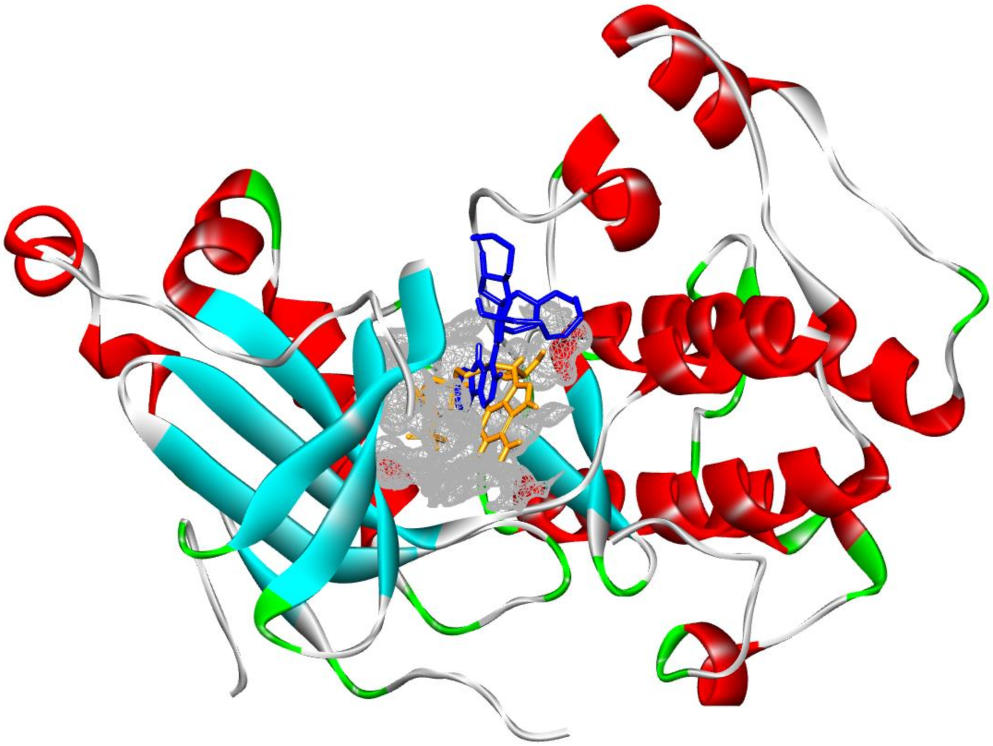

Figure S16: MZA (blue) and ATP (orange) is shown docked to protein kinase C (binding pocket is shown as gray mesh) (PKCα) (PDB ID: 3IW4)

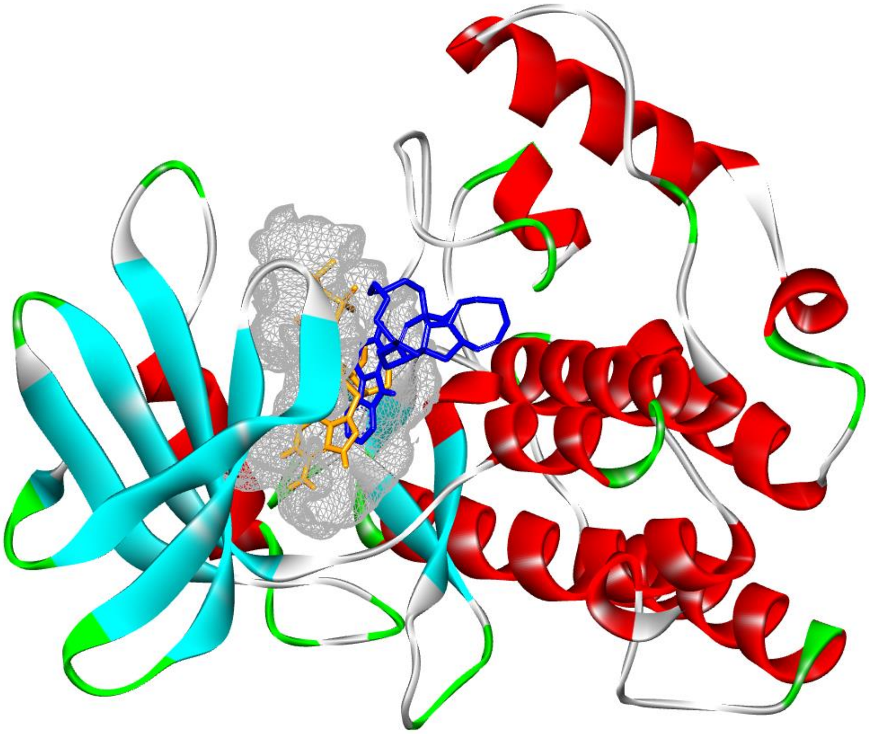

Figure S17: MZA (blue) and ATP (orange) is shown docked to casein kinase-1 (binding pocket is shown as gray mesh) (CK1) (PDB ID: 1CSN)

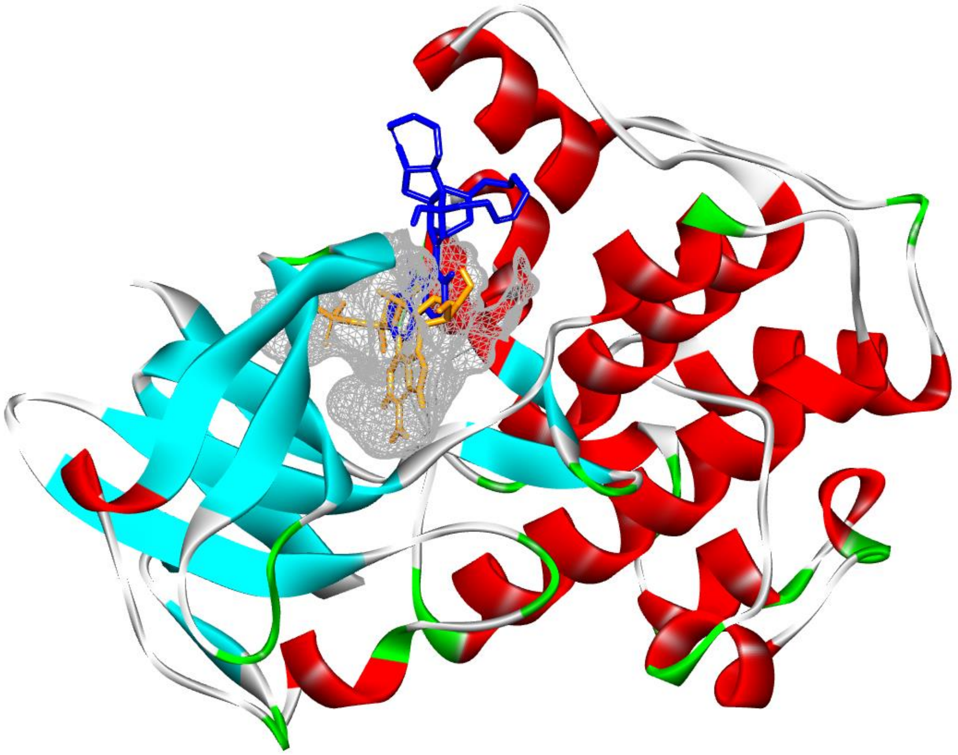

Figure S18: MZA (blue) and ATP (orange) is shown docked to MAPK-activated protein kinase-2 (binding pocket is shown as gray mesh) (MAPKAP-K2 (RSK-2)) (PDB ID: 3G51)

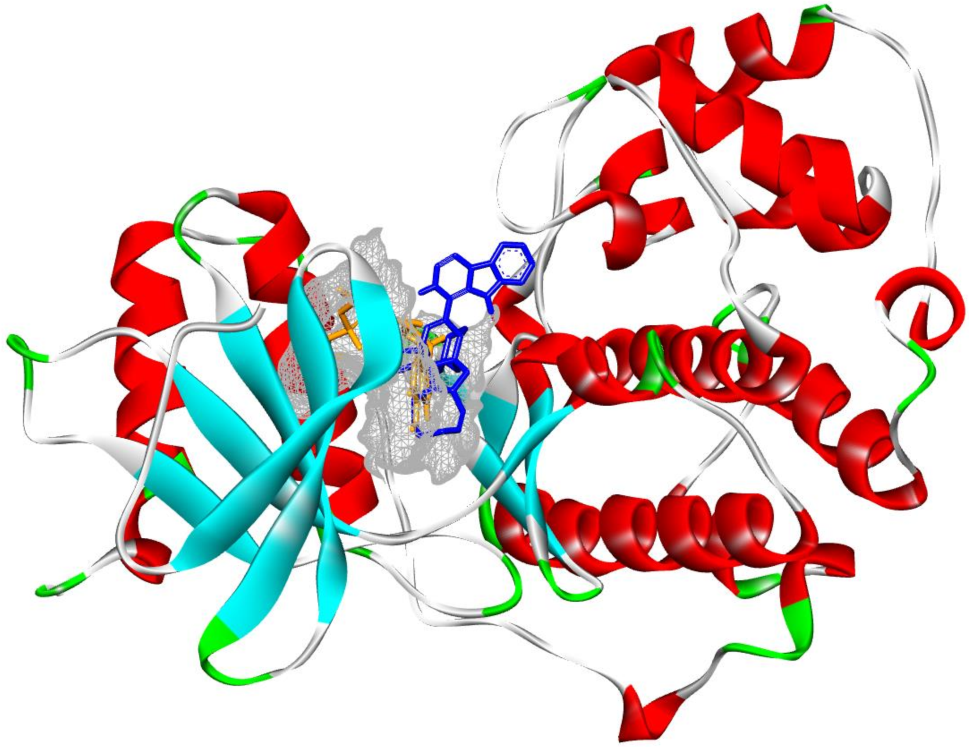

Figure S19: MZA (blue) and ATP (orange) is shown docked to mitogen-activated protein kinase (binding pocket is shown as gray mesh) (MAPK2/ERK2) (PDB ID: 2OJJ)

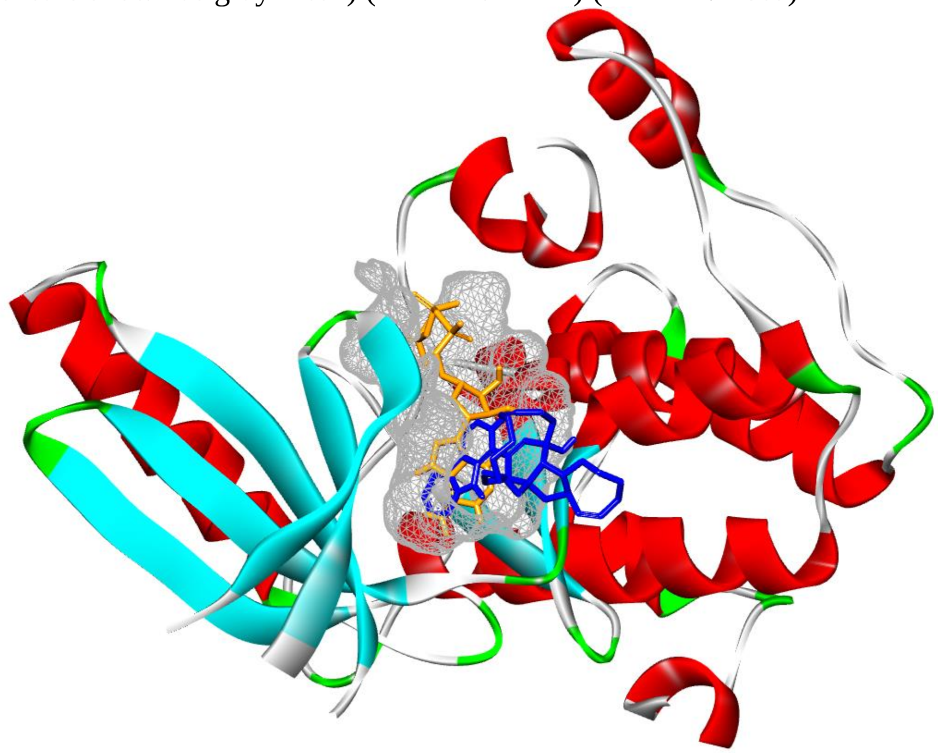

Figure S20: MZA (blue) and ATP (orange) is shown docked to NIMA-related protein kinase 2α (binding pocket is shown as gray mesh) (NEK2α) (PDB ID: 5M53)

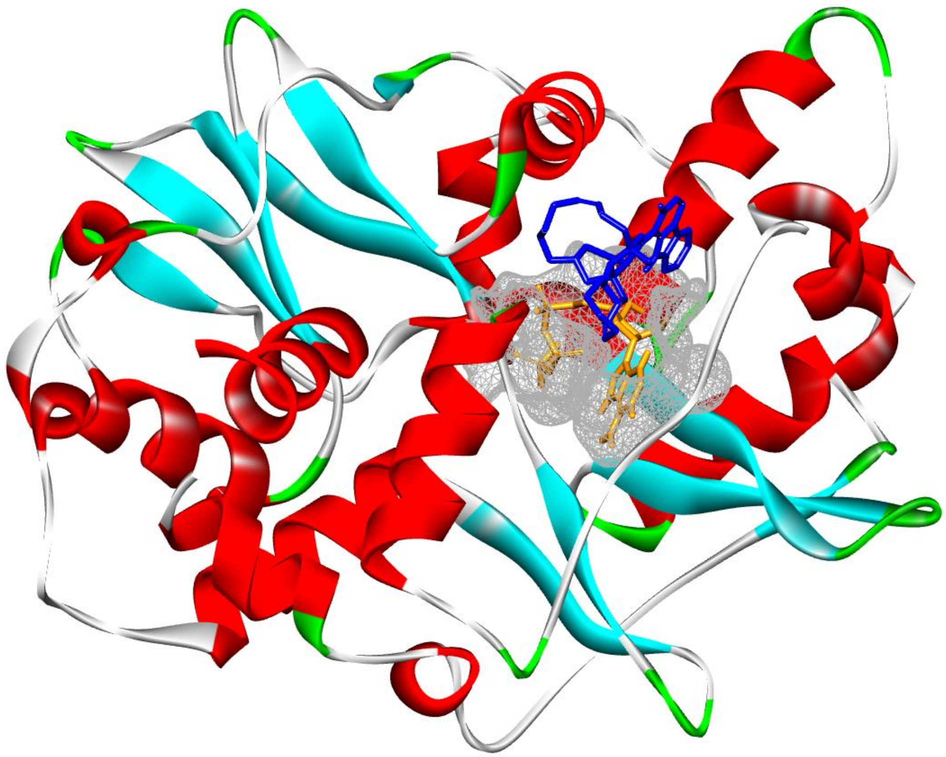

Figure S21: MZA (blue) and ATP (orange) is shown docked to AMP-activated protein kinase (binding pocket is shown as gray mesh) (AMPK) (PDB ID: 2YA3)

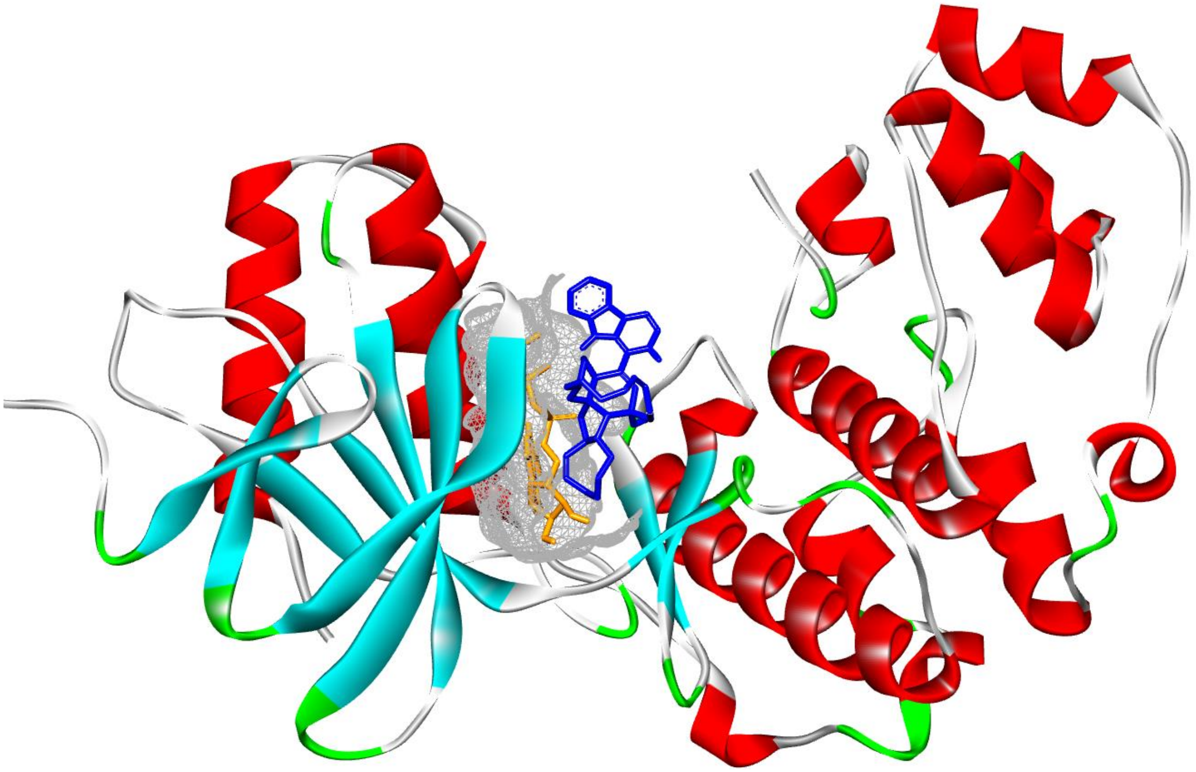

Figure S22: MZA (blue) and ATP (orange) is shown docked to stress-activated protein kinase-4 (binding pocket is shown as gray mesh) (SAPK4/P38δ) (PDB ID: 5ekn)

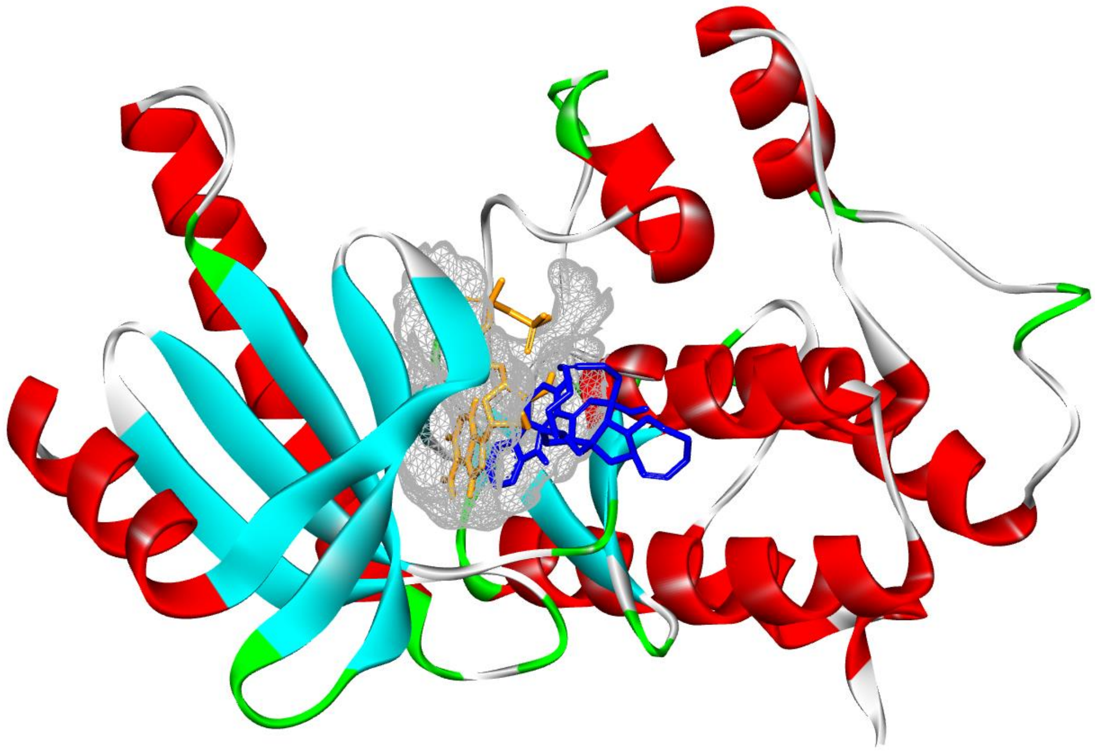

Figure S23: MZA (blue) and ATP (orange) is shown docked to phosphorylase kinase (binding pocket is shown as gray mesh) (PHK) (PDB ID: 2Y7J)

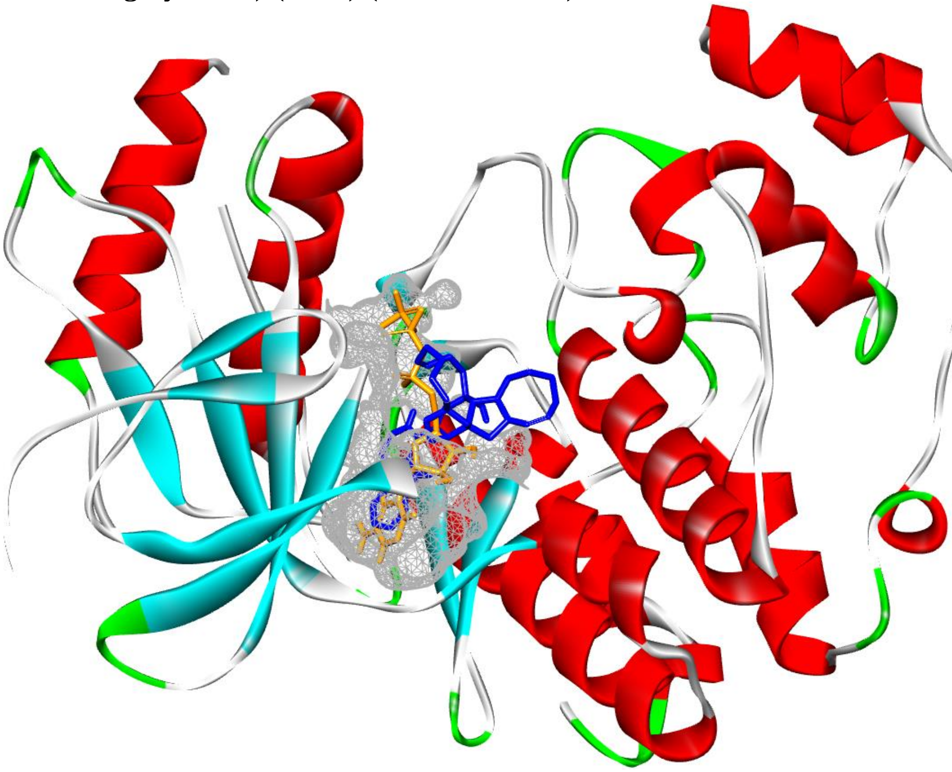

Figure S24: MZA (blue) and ATP (orange) is shown docked to stress-activated protein kinase-3 (binding pocket is shown as gray mesh) (SAPK3/P38γ) (PDB ID: 1CM8)

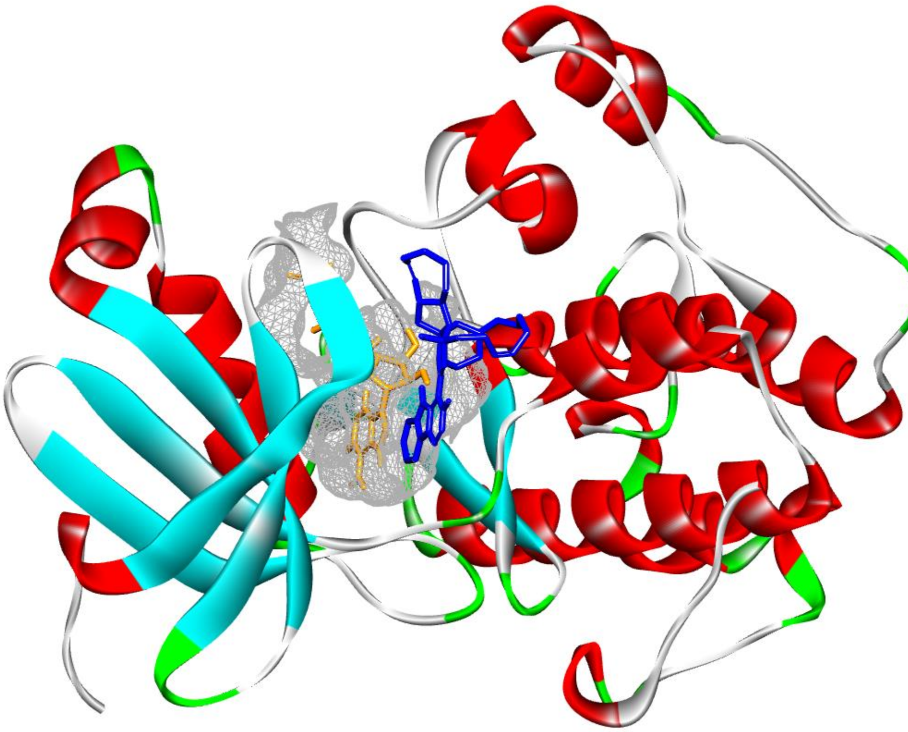

Figure S25: MZA (blue) and ATP (orange) is shown docked to 3-phosphoinositide-dependent protein kinase-1 (binding pocket is shown as gray mesh) (PDK1) (PDB ID: 5LVL)

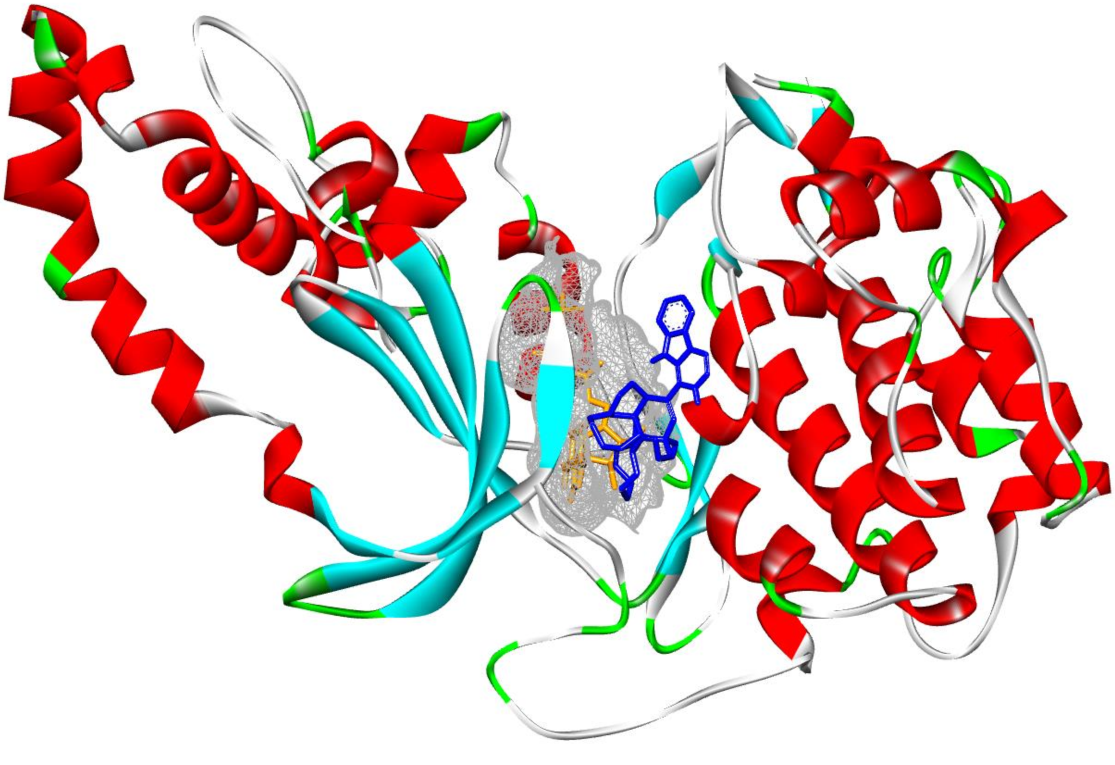

Figure S26: MZA (blue) and ATP (orange) is shown docked to Rho-dependent protein kinase (binding pocket is shown as gray mesh) (ROCK-II) (PDB ID: 6ED6)

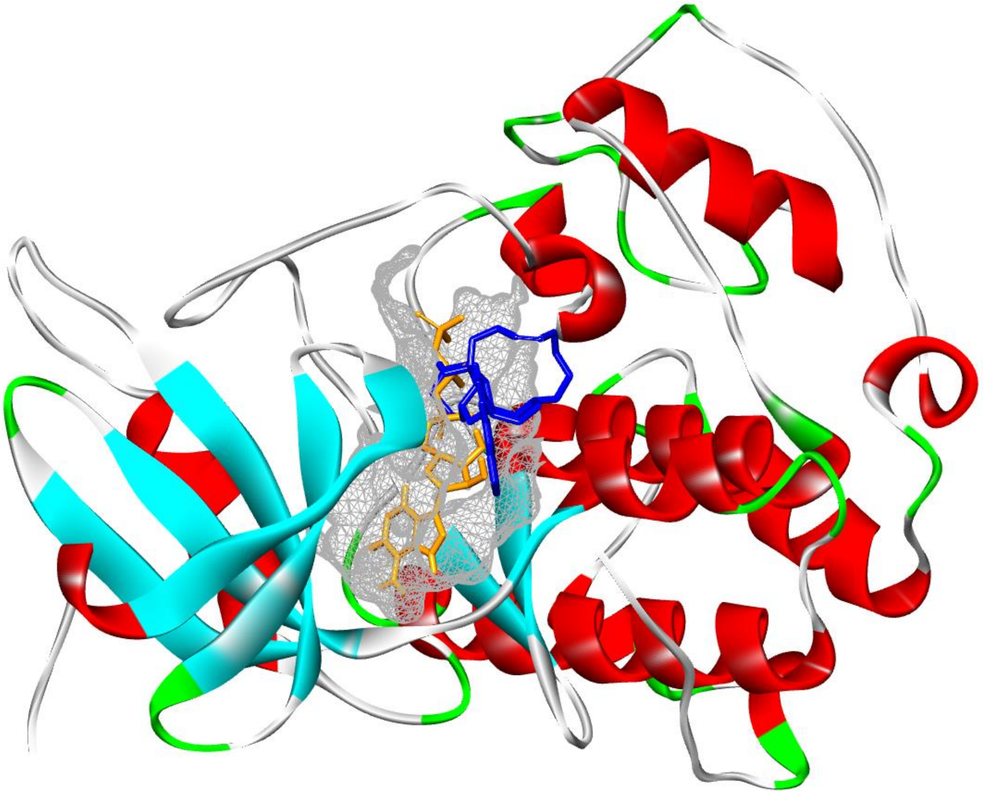

Figure S27: MZA (blue) and ATP (orange) is shown docked to cyclin-dependent kinase 2-cyclin A complex (binding pocket is shown as gray mesh) (CDK2/cyclin A) (PDB ID: 4EZ7)

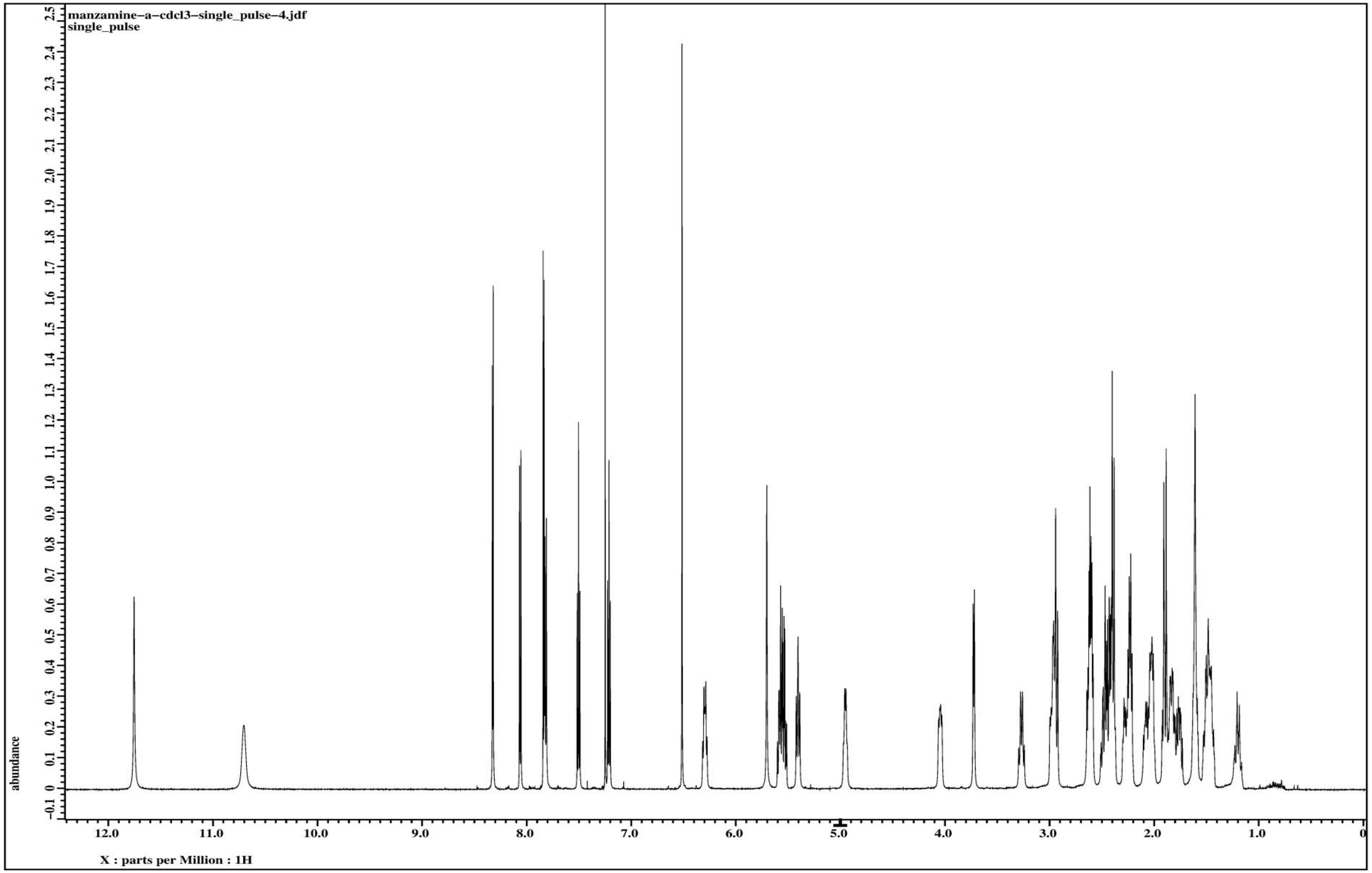

Figure S28. ^{1}H NMR of Manzamine A (CDCL3, 600 MHz)

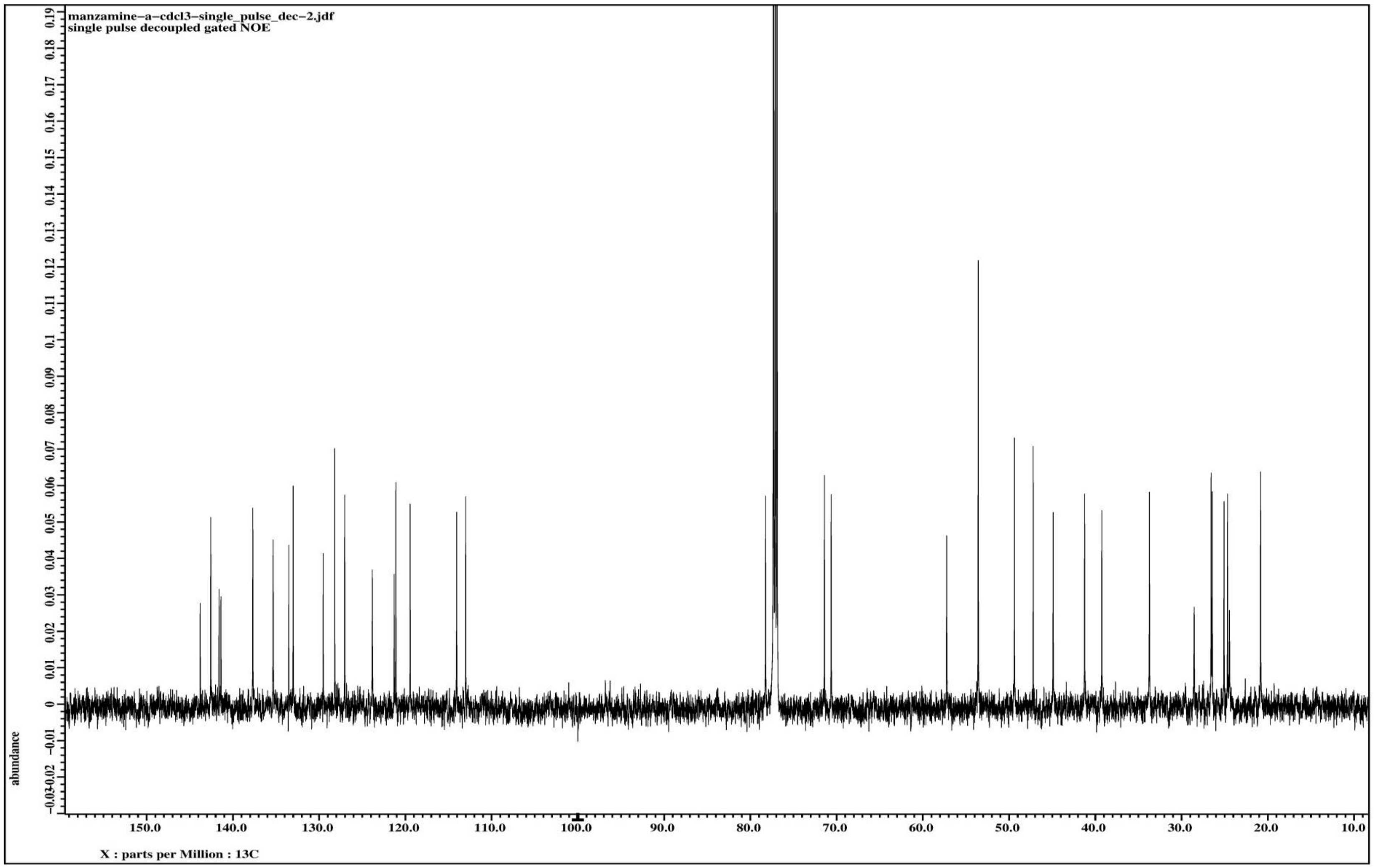

Figure S29. ^{13}C NMR of Manzamine A (CDCL3, 150 MHz)

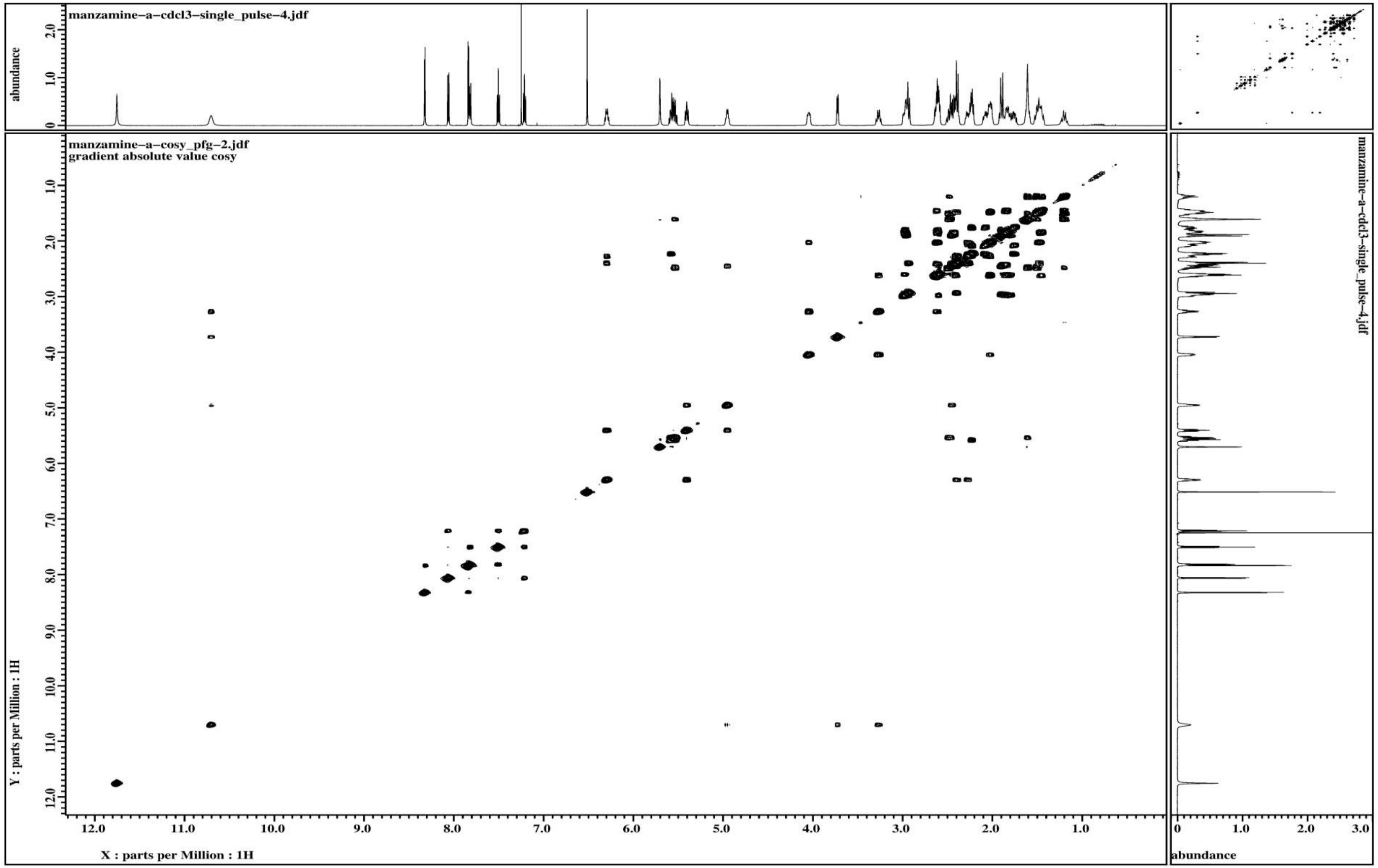

Figure S30. 2D-COSY Spectrum Manzamine A (CDCL3, 600 MHz)

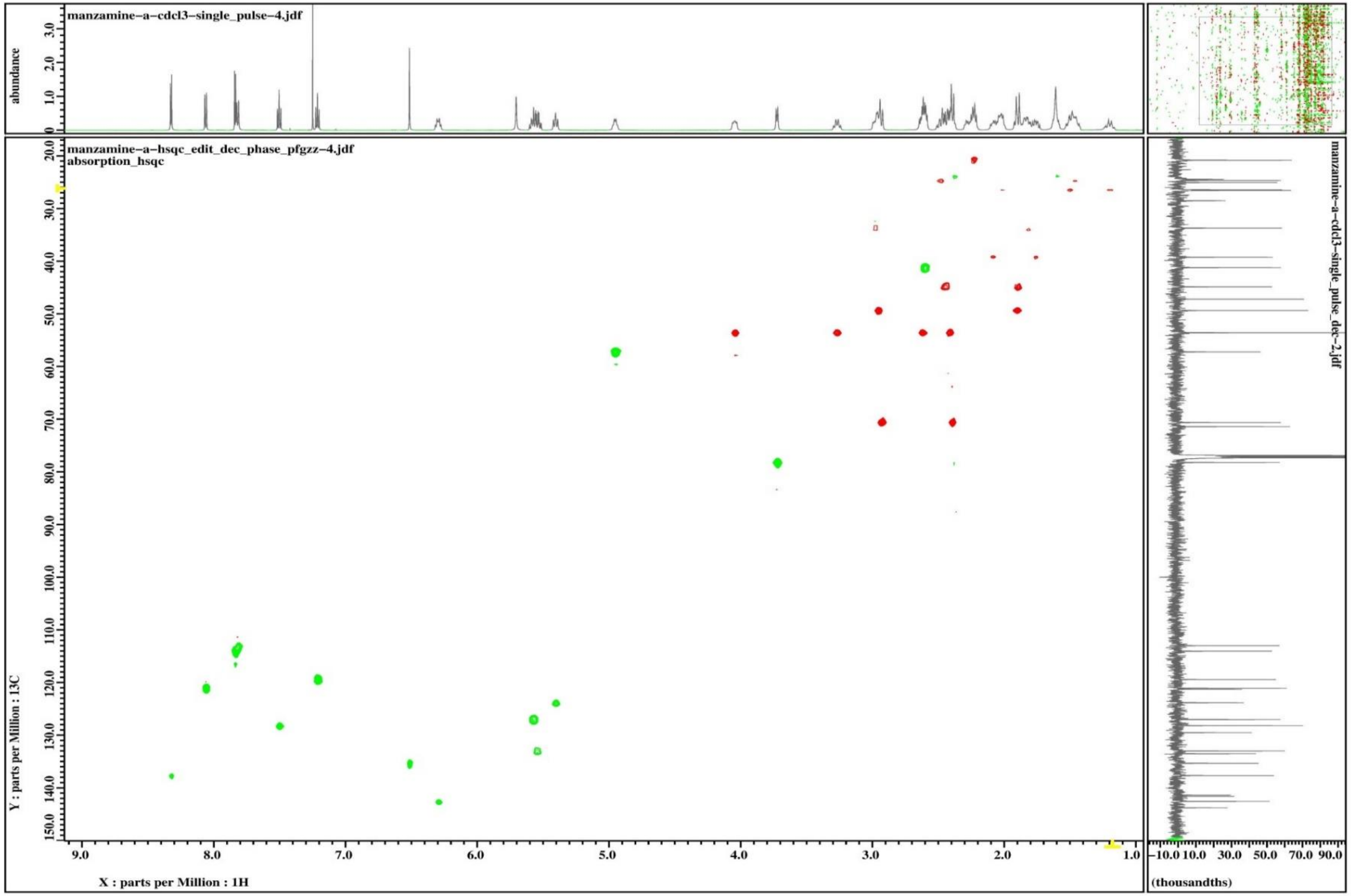

Figure S31. 2D edited g-HSQC Spectrum of Manzamine A (CDCL3, 600 MHz
